# Radical Cystectomy after BCG Immunotherapy for High-Risk Nonmuscle-Invasive Bladder Cancer in Patients with Previous Prostate Radiotherapy

**DOI:** 10.1155/2013/405064

**Published:** 2013-07-17

**Authors:** Manoj V. Rao, Marcus L. Quek, Gautam Jayram, Chandy Ellimoottil, Timothy Sondej, Cory M. Hugen, Robert C. Flanigan, Gary D. Steinberg

**Affiliations:** ^1^Department of Urology, Loyola University Medical Center, Room 261, Maywood, IL 60153, USA; ^2^Department of Surgery, Section of Urology, University of Chicago, 5841 South Maryland Avenue, MC6038 Chicago, IL 60637, USA

## Abstract

*Purpose*. Intravesical *Bacillus* Calmette-Guerin (BCG) immunotherapy is indicated for high-grade nonmuscle-invasive bladder cancer (NMIBC). The efficacy of BCG in patients with a history of previous pelvic radiotherapy (RT) may be diminished. We evaluated the outcomes of radical cystectomy for BCG-treated recurrent bladder cancer in patients with a history of RT for prostate cancer (PC). *Methods*. A retrospective chart review was performed to identify patients with primary NMIBC. We compared the outcomes of three groups of patients who underwent radical cystectomy for BCG-refractory NMIBC: those with a history of RT for PC, those who previously underwent radical prostatectomy (RP), and a cohort without PC or RT exposure. *Results*. From 1996 to 2008, 53 patients underwent radical cystectomy for recurrent NMIBC despite BCG. Those with previous pelvic RT were more likely to have a higher pathologic stage and decreased recurrence-free survival compared to the groups without prior RT exposure. *Conclusion.* Response rates for intravesical BCG therapy may be impaired in those with prior prostate radiotherapy. Patients with a history of RT who undergo radical cystectomy after failed BCG are more likely to be pathologically upstaged and have decreased recurrence-free survival. Earlier consideration of radical cystectomy may be warranted for those with NMIBC who previously received RT for PC.

## 1. Introduction

 In 2012, over 240,000 American men will be diagnosed with prostate cancer [1]. Approximately 28% will receive some form of radiation therapy (RT) [2]. Pelvic radiation may be associated with an elevated risk of secondary bladder malignancies that may be seen as early as five years after exposure [3]. 

Intravesical BCG therapy is a standard treatment for high-risk nonmuscle-invasive bladder cancer (NMIBC) (clinical stages Ta, Tis, and T1) [4]. We have previously shown that 50% of patients with NMIBC who were previously exposed to prostate RT will have a durable response to intravesical BCG. We now report our experience with radical cystectomy after failed BCG immunotherapy for high-risk NMIBC in men with and without a prior history of RT for PC. 

## 2. Methods

With institutional review board approval, we retrospectively identified all patients who underwent radical cystectomy for recurrent/persistent high-risk NMIBC urothelial carcinoma and received intravesical BCG therapy from a dataset of nearly 1500 cystectomy patients at two academic medical centers from 1995 to 2008. We divided this cohort into three groups based on the history of PC and its associated treatment modality: (1) those with a history of PC treated with RT, (2) those who underwent prior radical prostatectomy (RP) alone, and (3) those without PC treatment prior to radical cystectomy. The three groups were compared focusing on perioperative complications, histopathologic findings, and clinical outcomes. All patients who received brachytherapy and/or external beam radiotherapy as primary treatment or as adjuvant treatment following RP or as salvage for locally-recurrent PC were included in Group 1. Recurrence-free survival was determined by the Kaplan-Meier method. 

Statistical analyses were performed by means of Fisher's exact, chi square, *t*-test, ANOVA, and Kaplan-Meier methods using SPSS version 16 (SPSS, Chicago, IL, USA). All statistical analyses were 2 sided, and *P* < 0.05 was considered statistically significant.

## 3. Results

We identified 53 patients who underwent radical cystectomy for NMIBC after failed BCG therapy from two academic medical centers. Twelve had previous prostate RT (Group 1), 6 had a history of PC treated with RP alone (Group 2), and 35 had no history of PC or RT exposure (Group 3). Group 3 included six females. The clinical and perioperative characteristics of these patients are shown in Table 1. Patient age was significantly different between groups, with the irradiated group being older than the other groups (*P* = 0.01). Clinical stages were not significantly different between groups. The differences in transfusion rate and estimated blood loss were not statistically significant. 

Pathologic outcomes are listed in Table 2. Pathological upstaging (pT2–4 or pN1–3) was 58% in Group 1, 29% in Group 2, and 20% in Group 3 (*P* = 0.01). One patient in Group 1 was pT1N1M0. Extravesical extension (≥pT3) was also significantly higher in Group 1 versus Group 3 patients (*P* < 0.02). The incidence of carcinoma in situ in pathological specimens was highest in Group 3 (*P* < 0.001). The number of positive margins was not statistically different between the three groups; however, local recurrences (defined as pelvic soft tissue or urethra) were highest in Group 1 (42%). Residual prostate cancer was identified in two patients in Group 1 and one patient in Group 2. One patient from Group 1 required androgen deprivation therapy due to recurrent prostate cancer after cystectomy. 

Postoperative complications and associated interventions are listed in Table 3. Intraoperative complications included a rectal injury in Group 1 and an obturator nerve injury in Group 3. The rate of ureteroenteric obstruction requiring nephrostomy tube placement was also higher in Group 1 with a total of 6 kidneys out of 24 requiring temporary drainage (*P* = 0.001). In Group 1 nephrostomy tubes were placed in three patients for anastomotic strictures and one patient due to upper tract urothelial malignancy. In Group 3, nephrostomy tube drainage was required for two patients, one with an anastomotic stricture and the other with an upper tract recurrence causing obstruction. Of the patients that were initially managed with nephrostomy tube drainage, only one patient in Group 3 required antegrade incision and dilation of an anastomotic stricture. In the remaining patients, the nephrostomies were removed without further sequelae, and no further intervention was required at the last follow-up. The majority of complications occurring in Group 2 were related to urinary continence issues with patients requiring urethrotomy or artificial urinary sphincter placement. Postoperative Clavien major and minor complications were not significantly different, and there were no perioperative mortalities within 30 days of surgery.

**Figure 1:**
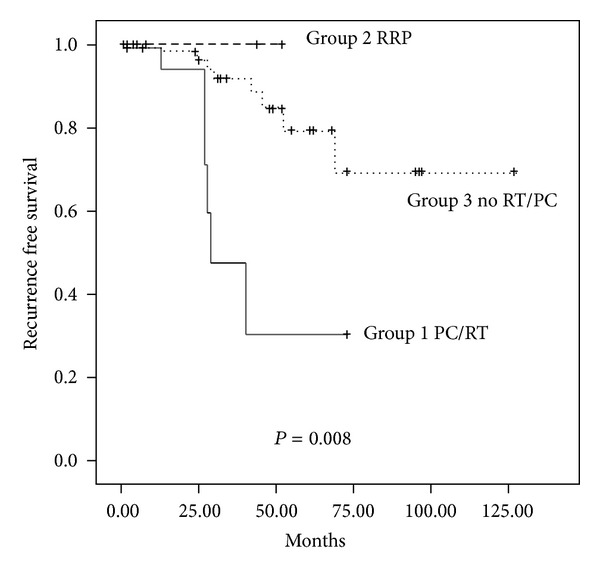
Recurrence-free survival for patients treated with radical cystectomy stratified by BCG failure group.

Recurrence-free survival was significantly worse for the patients with previous prostate RT (42%) compared to the group with previous prostatectomy (100%) and those with no history of prostate cancer or RT (71%) (*P* < 0.01; Figure 1). The median followup was 29 months (IQR 8–52 months). 

## 4. Discussion

 We previously reported that the efficacy of BCG may be diminished after PC RT with only 50% showing a durable response to intravesical therapy [5]. As such, we sought to assess the outcomes of the patients with high-risk NMIBC refractory to intravesical BCG that underwent radical cystectomy. To our knowledge, this is the first study to specifically analyze the outcomes of cystectomy for BCG-refractory NMIBC in patients with prior prostate RT. In the present series, those patients with a history of prior prostate RT were significantly more likely to be clinically understaged and more importantly demonstrated a higher risk for disease recurrence following radical cystectomy than those without previous exposure to pelvic radiation. 

Other studies have similarly shown that patients who undergo radical cystectomy after RT for PC have a high rate of nonorgan-confined disease, between 50–60% [3, 6]. The prevalence of pathological upstaging seen in the cystectomy cohort with prior RT is concerning. In the present study, despite the shorter follow-up period, the majority of patients with prior RT who underwent cystectomy for failed BCG therapy recurred primarily within the pelvis. Whether “earlier” consideration of radical cystectomy in the setting of nonmuscle-invasive bladder cancer and previous prostate radiotherapy would improve these outcomes remains to be determined. In addition, improvement in initial radiologic staging as well as subsequent imaging at the time of disease recurrence is of paramount importance. It is likely that the burden of disease in the patients who underwent cystectomy was significantly underestimated at the time of the initial diagnosis of urothelial carcinoma of the bladder. 

Patients who undergo radical cystectomy after previous PC/RT have a higher rate of complications. Eisenberg et al. reported a 21.1% rate of major urinary diversion-related complications in a cohort of salvage cystectomy patients [7]. Ramani et al. demonstrated a higher stricture rate in patients treated with primary radiotherapy for pelvic malignancy and then undergone salvage cystectomy [8]. Our higher rate of postoperative nephrostomy drainage in the RT group indicates that RT may be a risk factor for ureteroenteric anastomotic obstruction, likely a result of ischemia secondary to effects on the microcirculation of the distal ureter and/or bowel. Resection of the ureter more proximally (above the common iliac bifurcation) in patients with previous pelvic radiotherapy may help prevent ureteroenteric strictures [9].

This study has several limitations that should be addressed. It is a small retrospective study of patients treated at 2 academic medical centers over a 13-year period. Both centers have tertiary referral practices, and thus the patients treated may be at higher risk than would be seen in the community setting. Therefore, we may be overestimating the problem of clinical understaging at the time of cystectomy in the post-RT patients. Furthermore, the utilization of newer modalities of radiotherapy, such as intensity modulated radiotherapy, has changed over the last decade which may impact our findings [10]. Although our sample size is small, it adds to the other prior small published series evaluating radical cystectomy outcomes of patients with prior prostate RT [11, 12].

## 5. Conclusions

Intravesical BCG therapy may not be as effective for nonmuscle-invasive bladder cancer in the setting of prior prostate radiotherapy as evidenced by the high rate of pathologic upstaging. Radiation-induced bladder cancer may represent a more aggressive phenotype for which early consideration for radical cystectomy should be given. 

## Figures and Tables

**Table 1:** Demographics of patients treated with radical cystectomy for BCG failure stratified by previous prostate radiotherapy exposure.

	Group 1 (RT)	Group 2 (RP)	Group 3 (no PC/RT)	*P*-value
No. of patients	12	6	35	
Mean age	72.9 ± 4.69	66.9 ± 5.18	64.2 ± 10.4	0.0065
Clinical stage				0.47
CIS	5 (42)	1 (17)	8 (23)	
Ta	1 (8)	1 (17)	4 (11)	
T1	6 (50)	4 (66)	23 (66)	
Any CIS features preop	5 (42)	2 (33)	21 (60)	0.33
Diversion type				
Ileal conduit	8 (66)	1 (17)	13 (22)	0.09
Continent diversion	4 (33)	5 (83)	22 (63)	
Orthotopic neobladder	0	5 (83)	17 (49)	
Indiana pouch	4 (33)	0	5 (14)	
Estimated blood loss (mL)	683 ± 326	640 ± 169	789 ± 522	0.65
Intraoperative complication	1 (8)	0	1 (3)	0.61
Transfusion	1 (8)	0	9 (26)	0.19
Time to RC from PC diagnosis (years)	6.2 ± 4.3	5.5 ± 2.0		0.68

Numbers represent mean ± SD or no. (%) as appropriate.

RC: radical cystectomy.

**Table 2:** Pathological and clinical outcomes of radical cystectomy following BCG failure.

	Group 1 (RT)	Group 2 (RP)	Group 3 (no PC/RT)	*P*-value
Pathological stage				
pT0	2 (17)	1 (17)	2 (6)	0.36
pTis	1 (8)	1 (17)	9 (26)	
pTa	0	0	3 (9)	
pT1	2 (17)	2 (33)	14 (40)	
pT2	3 (25)	0	4 (11)	
pT3	3 (25)	2 (33)	2 (6)	
pT4	1 (8)	0	1 (3)	
Any CIS in final pathologic specimen	3 (25)	2 (33)	30 (86)	<0.001
Upstaged (≥T2 or N+)	8 (58)	2 (33)	7 (20)	0.01
Node-positive	2 (33)	0	1 (3)	0.17
Positive Margin	2 (17)	0	3 (9)	0.43
No. of pts with recurrence	6 (58)	0	10 (29)	0.09
Local (urethra, pelvis)	5 (42)	0	4 (11)	0.028
Pelvic soft tissue	3	0	2	
Urethra	2	0	2	
Urothelial (ureter/upper tract)	2 (17)	0	8 (23)	0.65
Distant	0	0	1 (3)	0.55

Numbers represent mean ± SD or no. (%) as appropriate.

**Table 3:** Postcystectomy complications stratified by BCG failure group.

	Group 1 (RT)	Group 2 (RP)	Group 3 (no PC/RT)	*P*-value
No. of pts requiring nephrostomy tube	4 (33)	0	2 (6)	0.001
Ureteroenteric stricture	3	0	1	
Upper tract malignancy	1	0	1	
No. of pts requiring secondary operations	8 (67)	2 (33)	12 (34)	0.13
Number of operations	7	7	12	
Urologic surgery	6 (50)	2 (33)	8 (23)	0.20
DVIU	0	6	0	
AUS	0	1	0	
Nephroureterectomy	1	0	4	
Urethrectomy	2	0	1	
Biopsy	3	0	0	
Upper tract tumor fulguration	0	0	2	
Nonurologic surgery	6 (50)	0	6 (17)	0.30
Colostomy	1	0	0	
Laparotomy	2	0	4	
Parastomal hernia repair	1	0	1	
IVC filter	1	0	0	
Drainage of fluid collection	1	0	0	
No. of pts with any complication	10 (83)	2 (33)	20 (57)	0.99
No. of pts with Clavien major complication	6 (50)	2 (33)	7 (47)	0.13
No. of pts with Clavien minor complication	8 (67)	2 (33)	15 (43)	0.28

Numbers represent mean ± SD or no. (%) as appropriate.

DVIU: direct vision internal urethrotomy.

AUS: artificial urinary sphincter.
